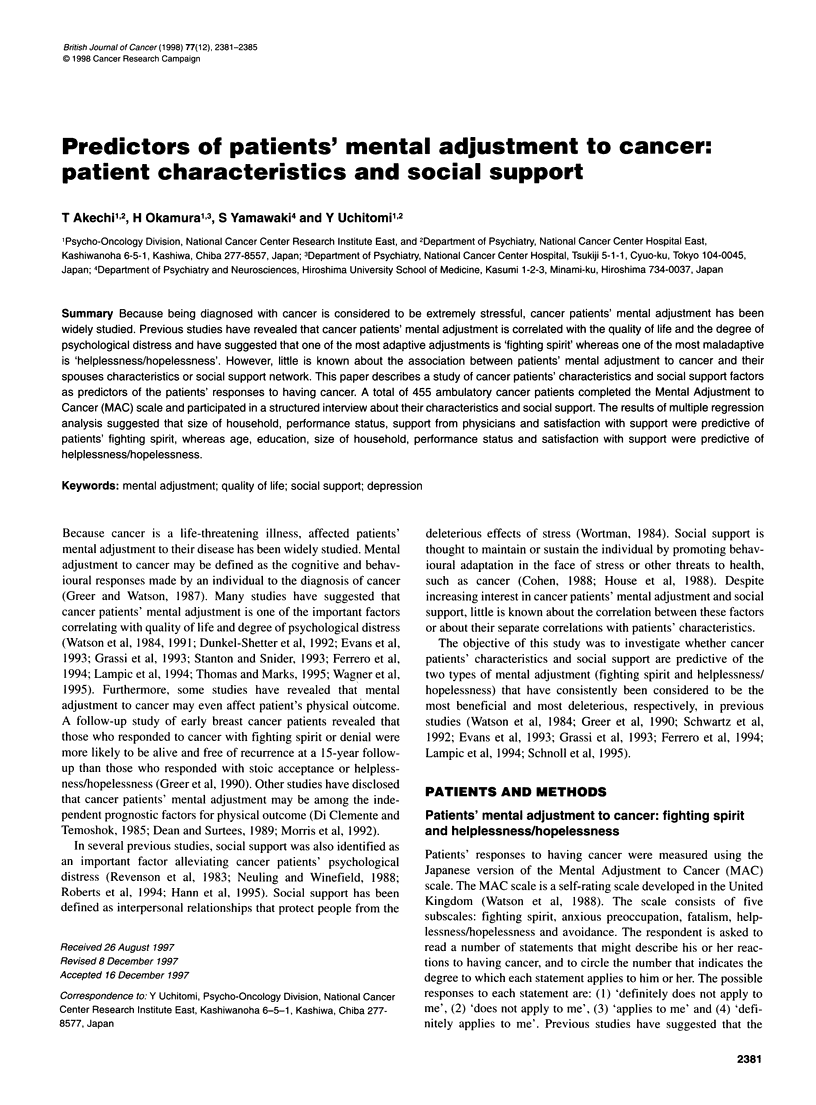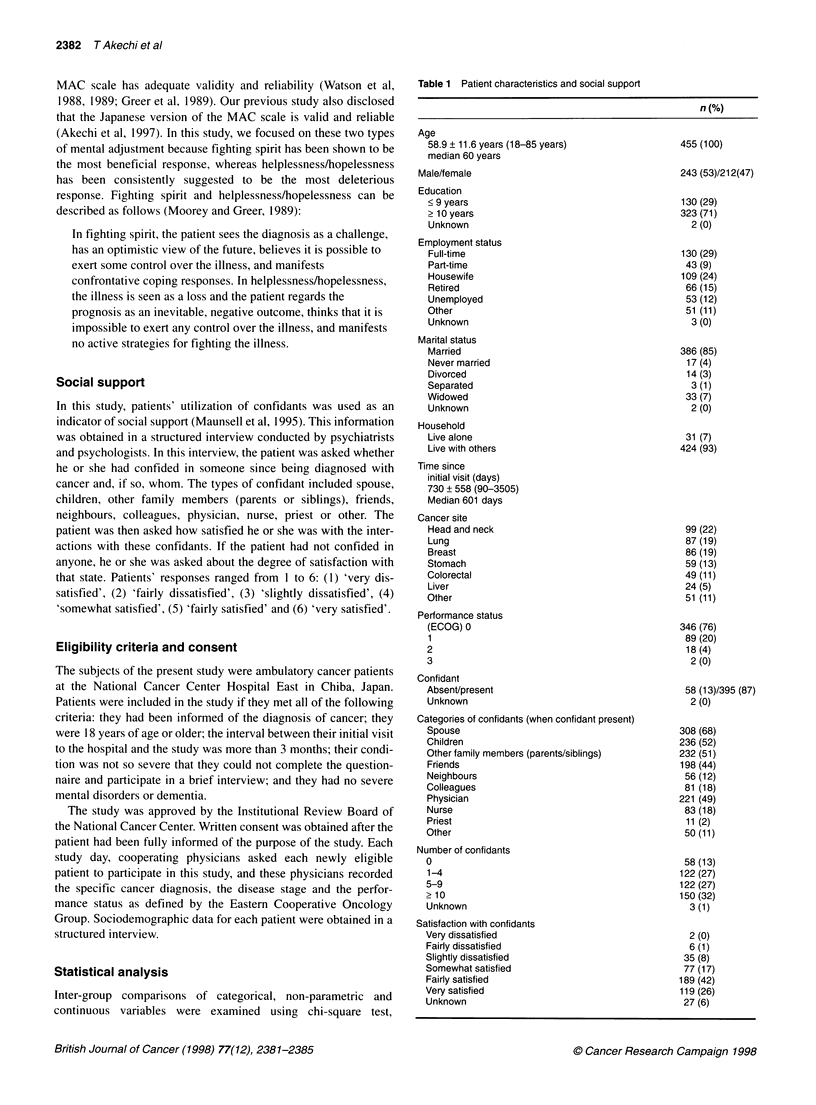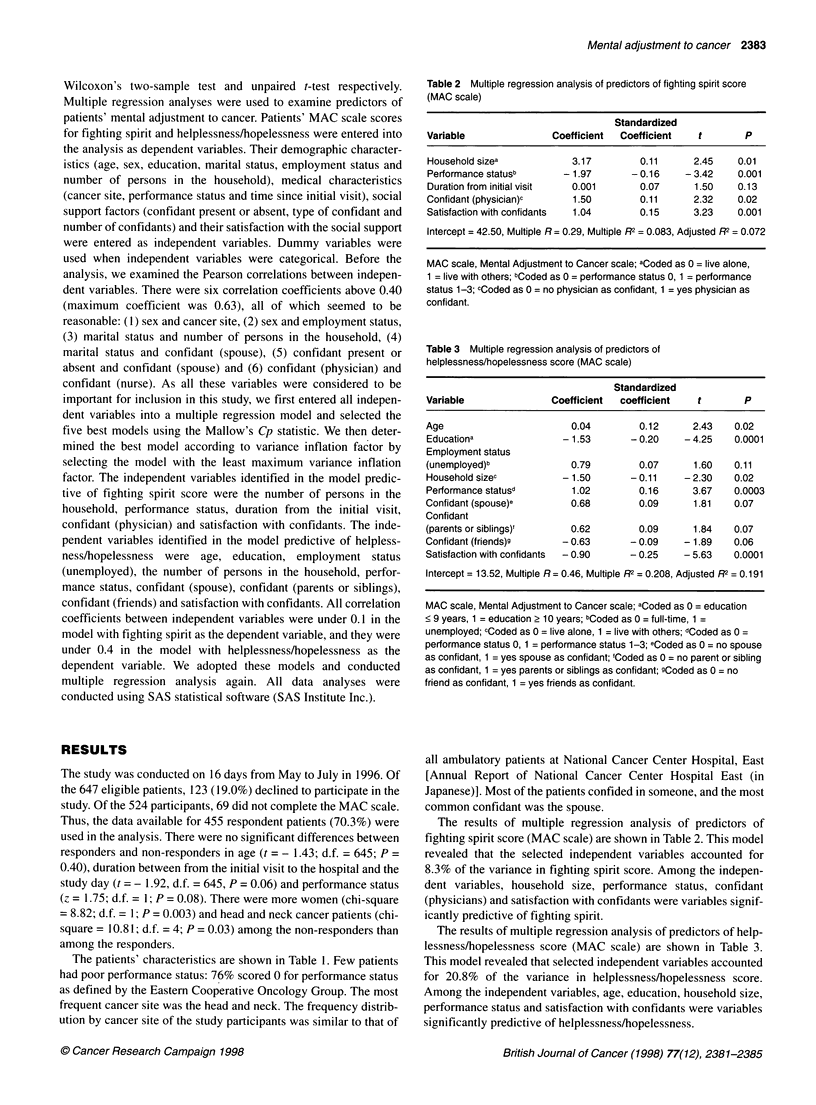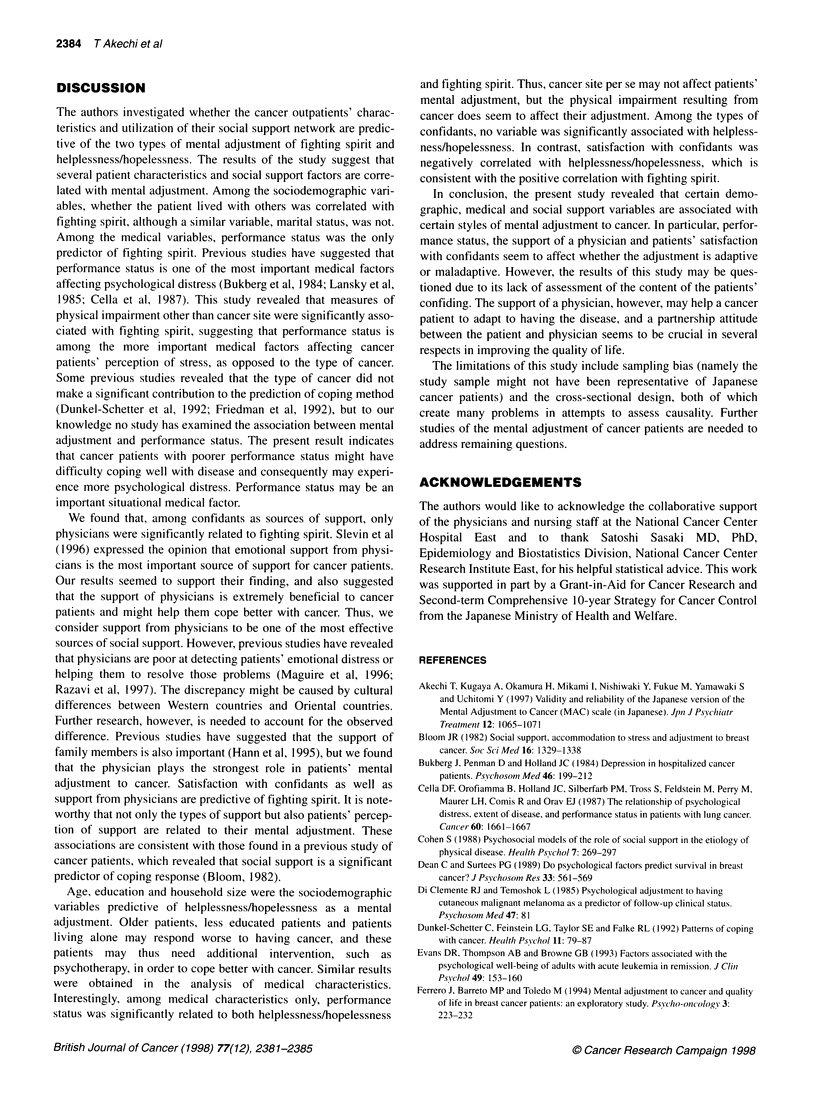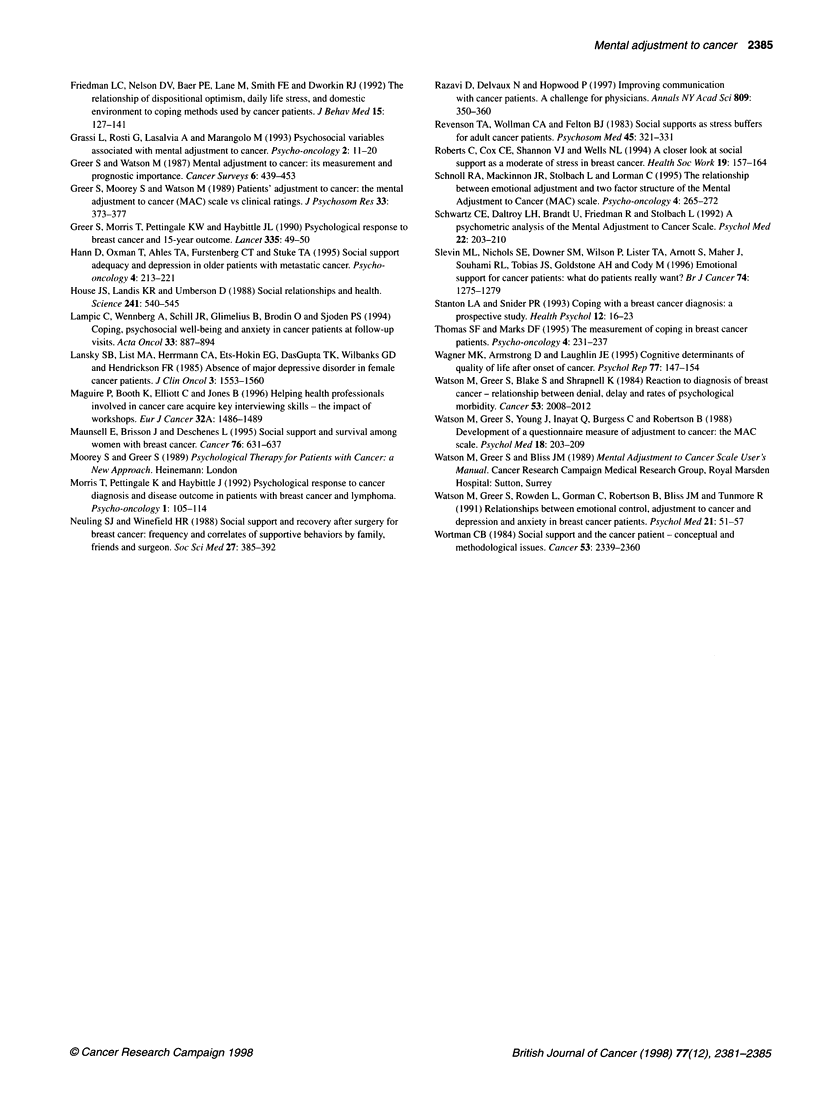# Predictors of patients' mental adjustment to cancer: patient characteristics and social support.

**DOI:** 10.1038/bjc.1998.396

**Published:** 1998-06

**Authors:** T. Akechi, H. Okamura, S. Yamawaki, Y. Uchitomi

**Affiliations:** Psycho-Oncology Division, National Cancer Center Research Institute East, Kashiwa, Chiba, Japan.

## Abstract

Because being diagnosed with cancer is considered to be extremely stressful, cancer patients' mental adjustment has been widely studied. Previous studies have revealed that cancer patients' mental adjustment is correlated with the quality of life and the degree of psychological distress and have suggested that one of the most adaptive adjustments is 'fighting spirit' whereas one of the most maladaptive is 'helplessness/hopelessness'. However, little is known about the association between patients' mental adjustment to cancer and their spouses characteristics or social support network. This paper describes a study of cancer patients' characteristics and social support factors as predictors of the patients' responses to having cancer. A total of 455 ambulatory cancer patients completed the Mental Adjustment to Cancer (MAC) scale and participated in a structured interview about their characteristics and social support. The results of multiple regression analysis suggested that size of household, performance status, support from physicians and satisfaction with support were predictive of patients' fighting spirit, whereas age, education, size of household, performance status and satisfaction with support were predictive of helplessness/hopelessness.


British Journal of Cancer (1998) 77(12), 2381–2385
© 1998 Cancer Research Campaign

### Predictors of patients' mental adjustment to cancer: patient characteristics and social support

**T Akechi[1,2], H Okamura[1,3], S Yamawaki[4] and Y Uchitomi[1,2]**

[1]Psycho-Oncology Division, National Cancer Center Research Institute East, and [2]Department of Psychiatry, National Cancer Center Hospital East, Kashiwanoha 6-5-1, Kashiwa, Chiba 277-8557, Japan; [3]Department of Psychiatry, National Cancer Center Hospital, Tsukiji 5-1-1, Cyuo-ku, Tokyo 104-0045, Japan; [4]Department of Psychiatry and Neurosciences, Hiroshima University School of Medicine, Kasumi 1-2-3, Minami-ku, Hiroshima 734-0037, Japan

**Summary** Because being diagnosed with cancer is considered to be extremely stressful, cancer patients' mental adjustment has been widely studied. Previous studies have revealed that cancer patients' mental adjustment is correlated with the quality of life and the degree of psychological distress and have suggested that one of the most adaptive adjustments is 'fighting spirit' whereas one of the most maladaptive is 'helplessness/hopelessness'. However, little is known about the association between patients' mental adjustment to cancer and their spouses characteristics or social support network. This paper describes a study of cancer patients' characteristics and social support factors as predictors of the patients' responses to having cancer. A total of 455 ambulatory cancer patients completed the Mental Adjustment to Cancer (MAC) scale and participated in a structured interview about their characteristics and social support. The results of multiple regression analysis suggested that size of household, performance status, support from physicians and satisfaction with support were predictive of patients' fighting spirit, whereas age, education, size of household, performance status and satisfaction with support were predictive of helplessness/hopelessness.

**Keywords:** mental adjustment; quality of life; social support; depression

Because cancer is a life-threatening illness, affected patients' mental adjustment to their disease has been widely studied. Mental adjustment to cancer may be defined as the cognitive and behavioural responses made by an individual to the diagnosis of cancer (Greer and Watson, 1987). Many studies have suggested that cancer patients' mental adjustment is one of the important factors correlating with quality of life and degree of psychological distress (Watson et al, 1984, 1991; Dunkel-Shetter et al, 1992; Evans et al, 1993; Grassi et al, 1993; Stanton and Snider, 1993; Ferrero et al, 1994; Lampic et al, 1994; Thomas and Marks, 1995; Wagner et al, 1995). Furthermore, some studies have revealed that mental adjustment to cancer may even affect patient's physical outcome. A follow-up study of early breast cancer patients revealed that those who responded to cancer with fighting spirit or denial were more likely to be alive and free of recurrence at a 15-year follow-up than those who responded with stoic acceptance or helplessness/hopelessness (Greer et al, 1990). Other studies have disclosed that cancer patients' mental adjustment may be among the independent prognostic factors for physical outcome (Di Clemente and Temoshok, 1985; Dean and Surtees, 1989; Morris et al, 1992).

In several previous studies, social support was also identified as an important factor alleviating cancer patients' psychological distress (Revenson et al, 1983; Neuling and Winefield, 1988; Roberts et al, 1994; Hann et al, 1995). Social support has been defined as interpersonal relationships that protect people from the deleterious effects of stress (Wortman, 1984). Social support is thought to maintain or sustain the individual by promoting behavioural adaptation in the face of stress or other threats to health, such as cancer (Cohen, 1988; House et al, 1988). Despite increasing interest in cancer patients' mental adjustment and social support, little is known about the correlation between these factors or about their separate correlations with patients' characteristics.

The objective of this study was to investigate whether cancer patients' characteristics and social support are predictive of the two types of mental adjustment (fighting spirit and helplessness/hopelessness) that have consistently been considered to be the most beneficial and most deleterious, respectively, in previous studies (Watson et al, 1984; Greer et al, 1990; Schwartz et al, 1992; Evans et al, 1993; Grassi et al, 1993; Ferrero et al, 1994; Lampic et al, 1994; Schnoll et al, 1995).

*Received 26 August 1997*
*Revised 8 December 1997*
*Accepted 16 December 1997*

*Correspondence to:* Y Uchitomi, Psycho-Oncology Division, National Cancer Center Research Institute East, Kashiwanoha 6–5–1, Kashiwa, Chiba 277-8577, Japan

### PATIENTS AND METHODS

### Patients' mental adjustment to cancer: fighting spirit and helplessness/hopelessness

Patients' responses to having cancer were measured using the Japanese version of the Mental Adjustment to Cancer (MAC) scale. The MAC scale is a self-rating scale developed in the United Kingdom (Watson et al, 1988). The scale consists of five subscales: fighting spirit, anxious preoccupation, fatalism, helplessness/hopelessness and avoidance. The respondent is asked to read a number of statements that might describe his or her reactions to having cancer, and to circle the number that indicates the degree to which each statement applies to him or her. The possible responses to each statement are: (1) 'definitely does not apply to me', (2) 'does not apply to me', (3) 'applies to me' and (4) 'definitely applies to me'. Previous studies have suggested that the

MAC scale has adequate validity and reliability (Watson et al, 1988, 1989; Greer et al, 1989). Our previous study also disclosed that the Japanese version of the MAC scale is valid and reliable (Akechi et al, 1997). In this study, we focused on these two types of mental adjustment because fighting spirit has been shown to be the most beneficial response, whereas helplessness/hopelessness has been consistently suggested to be the most deleterious response. Fighting spirit and helplessness/hopelessness can be described as follows (Moorey and Greer, 1989):

> In fighting spirit, the patient sees the diagnosis as a challenge, has an optimistic view of the future, believes it is possible to exert some control over the illness, and manifests confrontative coping responses. In helplessness/hopelessness, the illness is seen as a loss and the patient regards the prognosis as an inevitable, negative outcome, thinks that it is impossible to exert any control over the illness, and manifests no active strategies for fighting the illness.

### Social support

In this study, patients' utilization of confidants was used as an indicator of social support (Maunsell et al, 1995). This information was obtained in a structured interview conducted by psychiatrists and psychologists. In this interview, the patient was asked whether he or she had confided in someone since being diagnosed with cancer and, if so, whom. The types of confidant included spouse, children, other family members (parents or siblings), friends, neighbours, colleagues, physician, nurse, priest or other. The patient was then asked how satisfied he or she was with the interactions with these confidants. If the patient had not confided in anyone, he or she was asked about the degree of satisfaction with that state. Patients' responses ranged from 1 to 6: (1) 'very dissatisfied', (2) 'fairly dissatisfied', (3) 'slightly dissatisfied', (4) 'somewhat satisfied', (5) 'fairly satisfied' and (6) 'very satisfied'.

### Eligibility criteria and consent

The subjects of the present study were ambulatory cancer patients at the National Cancer Center Hospital East in Chiba, Japan. Patients were included in the study if they met all of the following criteria: they had been informed of the diagnosis of cancer; they were 18 years of age or older; the interval between their initial visit to the hospital and the study was more than 3 months; their condition was not so severe that they could not complete the questionnaire and participate in a brief interview; and they had no severe mental disorders or dementia.

The study was approved by the Institutional Review Board of the National Cancer Center. Written consent was obtained after the patient had been fully informed of the purpose of the study. Each study day, cooperating physicians asked each newly eligible patient to participate in this study, and these physicians recorded the specific cancer diagnosis, the disease stage and the performance status as defined by the Eastern Cooperative Oncology Group. Sociodemographic data for each patient were obtained in a structured interview.

### Statistical analysis

Inter-group comparisons of categorical, non-parametric and continuous variables were examined using chi-square test,

**Table 1** Patient characteristics and social support

| | *n* (%) |
|---|---|
| Age | |
| 58.9 ± 11.6 years (18–85 years) median 60 years | 455 (100) |
| Male/female | 243 (53)/212(47) |
| Education | |
| ≤ 9 years | 130 (29) |
| ≥ 10 years | 323 (71) |
| Unknown | 2 (0) |
| Employment status | |
| Full-time | 130 (29) |
| Part-time | 43 (9) |
| Housewife | 109 (24) |
| Retired | 66 (15) |
| Unemployed | 53 (12) |
| Other | 51 (11) |
| Unknown | 3 (0) |
| Marital status | |
| Married | 386 (85) |
| Never married | 17 (4) |
| Divorced | 14 (3) |
| Separated | 3 (1) |
| Widowed | 33 (7) |
| Unknown | 2 (0) |
| Household | |
| Live alone | 31 (7) |
| Live with others | 424 (93) |
| Time since initial visit (days) | |
| 730 ± 558 (90–3505) | |
| Median 601 days | |
| Cancer site | |
| Head and neck | 99 (22) |
| Lung | 87 (19) |
| Breast | 86 (19) |
| Stomach | 59 (13) |
| Colorectal | 49 (11) |
| Liver | 24 (5) |
| Other | 51 (11) |
| Performance status | |
| (ECOG) 0 | 346 (76) |
| 1 | 89 (20) |
| 2 | 18 (4) |
| 3 | 2 (0) |
| Confidant | |
| Absent/present | 58 (13)/395 (87) |
| Unknown | 2 (0) |
| Categories of confidants (when confidant present) | |
| Spouse | 308 (68) |
| Children | 236 (52) |
| Other family members (parents/siblings) | 232 (51) |
| Friends | 198 (44) |
| Neighbours | 56 (12) |
| Colleagues | 81 (18) |
| Physician | 221 (49) |
| Nurse | 83 (18) |
| Priest | 11 (2) |
| Other | 50 (11) |
| Number of confidants | |
| 0 | 58 (13) |
| 1–4 | 122 (27) |
| 5–9 | 122 (27) |
| ≥ 10 | 150 (32) |
| Unknown | 3 (1) |
| Satisfaction with confidants | |
| Very dissatisfied | 2 (0) |
| Fairly dissatisfied | 6 (1) |
| Slightly dissatisfied | 35 (8) |
| Somewhat satisfied | 77 (17) |
| Fairly satisfied | 189 (42) |
| Very satisfied | 119 (26) |
| Unknown | 27 (6) |

© Cancer Research Campaign 1998

Wilcoxon's two-sample test and unpaired *t*-test respectively. Multiple regression analyses were used to examine predictors of patients' mental adjustment to cancer. Patients' MAC scale scores for fighting spirit and helplessness/hopelessness were entered into the analysis as dependent variables. Their demographic characteristics (age, sex, education, marital status, employment status and number of persons in the household), medical characteristics (cancer site, performance status and time since initial visit), social support factors (confidant present or absent, type of confidant and number of confidants) and their satisfaction with the social support were entered as independent variables. Dummy variables were used when independent variables were categorical. Before the analysis, we examined the Pearson correlations between independent variables. There were six correlation coefficients above 0.40 (maximum coefficient was 0.63), all of which seemed to be reasonable: (1) sex and cancer site, (2) sex and employment status, (3) marital status and number of persons in the household, (4) marital status and confidant (spouse), (5) confidant present or absent and confidant (spouse) and (6) confidant (physician) and confidant (nurse). As all these variables were considered to be important for inclusion in this study, we first entered all independent variables into a multiple regression model and selected the five best models using the Mallow's *Cp* statistic. We then determined the best model according to variance inflation factor by selecting the model with the least maximum variance inflation factor. The independent variables identified in the model predictive of fighting spirit score were the number of persons in the household, performance status, duration from the initial visit, confidant (physician) and satisfaction with confidants. The independent variables identified in the model predictive of helplessness/hopelessness were age, education, employment status (unemployed), the number of persons in the household, performance status, confidant (spouse), confidant (parents or siblings), confidant (friends) and satisfaction with confidants. All correlation coefficients between independent variables were under 0.1 in the model with fighting spirit as the dependent variable, and they were under 0.4 in the model with helplessness/hopelessness as the dependent variable. We adopted these models and conducted multiple regression analysis again. All data analyses were conducted using SAS statistical software (SAS Institute Inc.).

**Table 2** Multiple regression analysis of predictors of fighting spirit score (MAC scale)

| Variable | Coefficient | Standardized Coefficient | *t* | *P* |
|---|---|---|---|---|
| Household size[a] | 3.17 | 0.11 | 2.45 | 0.01 |
| Performance status[b] | – 1.97 | – 0.16 | – 3.42 | 0.001 |
| Duration from initial visit | 0.001 | 0.07 | 1.50 | 0.13 |
| Confidant (physician)[c] | 1.50 | 0.11 | 2.32 | 0.02 |
| Satisfaction with confidants | 1.04 | 0.15 | 3.23 | 0.001 |
| Intercept = 42.50, Multiple $R$ = 0.29, Multiple $R^2$ = 0.083, Adjusted $R^2$ = 0.072 | | | | |

MAC scale, Mental Adjustment to Cancer scale; [a]Coded as 0 = live alone, 1 = live with others; [b]Coded as 0 = performance status 0, 1 = performance status 1–3; [c]Coded as 0 = no physician as confidant, 1 = yes physician as confidant.

**Table 3** Multiple regression analysis of predictors of helplessness/hopelessness score (MAC scale)

| Variable | Coefficient | Standardized coefficient | *t* | *P* |
|---|---|---|---|---|
| Age | 0.04 | 0.12 | 2.43 | 0.02 |
| Education[a] | – 1.53 | – 0.20 | – 4.25 | 0.0001 |
| Employment status (unemployed)[b] | 0.79 | 0.07 | 1.60 | 0.11 |
| Household size[c] | – 1.50 | – 0.11 | – 2.30 | 0.02 |
| Performance status[d] | 1.02 | 0.16 | 3.67 | 0.0003 |
| Confidant (spouse)[e] | 0.68 | 0.09 | 1.81 | 0.07 |
| Confidant (parents or siblings)[f] | 0.62 | 0.09 | 1.84 | 0.07 |
| Confidant (friends)[g] | – 0.63 | – 0.09 | – 1.89 | 0.06 |
| Satisfaction with confidants | – 0.90 | – 0.25 | – 5.63 | 0.0001 |
| Intercept = 13.52, Multiple $R$ = 0.46, Multiple $R^2$ = 0.208, Adjusted $R^2$ = 0.191 | | | | |

MAC scale, Mental Adjustment to Cancer scale; [a]Coded as 0 = education ≤ 9 years, 1 = education ≥ 10 years; [b]Coded as 0 = full-time, 1 = unemployed; [c]Coded as 0 = live alone, 1 = live with others; [d]Coded as 0 = performance status 0, 1 = performance status 1–3; [e]Coded as 0 = no spouse as confidant, 1 = yes spouse as confidant; [f]Coded as 0 = no parent or sibling as confidant, 1 = yes parents or siblings as confidant; [g]Coded as 0 = no friend as confidant, 1 = yes friends as confidant.

### RESULTS

The study was conducted on 16 days from May to July in 1996. Of the 647 eligible patients, 123 (19.0%) declined to participate in the study. Of the 524 participants, 69 did not complete the MAC scale. Thus, the data available for 455 respondent patients (70.3%) were used in the analysis. There were no significant differences between responders and non-responders in age ($t$ = – 1.43; d.f. = 645; $P$ = 0.40), duration between from the initial visit to the hospital and the study day ($t$ = – 1.92, d.f. = 645, $P$ = 0.06) and performance status ($z$ = 1.75; d.f. = 1; $P$ = 0.08). There were more women (chi-square = 8.82; d.f. = 1; $P$ = 0.003) and head and neck cancer patients (chi-square = 10.81; d.f. = 4; $P$ = 0.03) among the non-responders than among the responders.

The patients' characteristics are shown in Table 1. Few patients had poor performance status: 76% scored 0 for performance status as defined by the Eastern Cooperative Oncology Group. The most frequent cancer site was the head and neck. The frequency distribution by cancer site of the study participants was similar to that of all ambulatory patients at National Cancer Center Hospital, East [Annual Report of National Cancer Center Hospital East (in Japanese)]. Most of the patients confided in someone, and the most common confidant was the spouse.

The results of multiple regression analysis of predictors of fighting spirit score (MAC scale) are shown in Table 2. This model revealed that the selected independent variables accounted for 8.3% of the variance in fighting spirit score. Among the independent variables, household size, performance status, confidant (physicians) and satisfaction with confidants were variables significantly predictive of fighting spirit.

The results of multiple regression analysis of predictors of helplessness/hopelessness score (MAC scale) are shown in Table 3. This model revealed that selected independent variables accounted for 20.8% of the variance in helplessness/hopelessness score. Among the independent variables, age, education, household size, performance status and satisfaction with confidants were variables significantly predictive of helplessness/hopelessness.

© Cancer Research Campaign 1998

### DISCUSSION

The authors investigated whether the cancer outpatients' characteristics and utilization of their social support network are predictive of the two types of mental adjustment of fighting spirit and helplessness/hopelessness. The results of the study suggest that several patient characteristics and social support factors are correlated with mental adjustment. Among the sociodemographic variables, whether the patient lived with others was correlated with fighting spirit, although a similar variable, marital status, was not. Among the medical variables, performance status was the only predictor of fighting spirit. Previous studies have suggested that performance status is one of the most important medical factors affecting psychological distress (Bukberg et al, 1984; Lansky et al, 1985; Cella et al, 1987). This study revealed that measures of physical impairment other than cancer site were significantly associated with fighting spirit, suggesting that performance status is among the more important medical factors affecting cancer patients' perception of stress, as opposed to the type of cancer. Some previous studies revealed that the type of cancer did not make a significant contribution to the prediction of coping method (Dunkel-Schetter et al, 1992; Friedman et al, 1992), but to our knowledge no study has examined the association between mental adjustment and performance status. The present result indicates that cancer patients with poorer performance status might have difficulty coping well with disease and consequently may experience more psychological distress. Performance status may be an important situational medical factor.

We found that, among confidants as sources of support, only physicians were significantly related to fighting spirit. Slevin et al (1996) expressed the opinion that emotional support from physicians is the most important source of support for cancer patients. Our results seemed to support their finding, and also suggested that the support of physicians is extremely beneficial to cancer patients and might help them cope better with cancer. Thus, we consider support from physicians to be one of the most effective sources of social support. However, previous studies have revealed that physicians are poor at detecting patients' emotional distress or helping them to resolve those problems (Maguire et al, 1996; Razavi et al, 1997). The discrepancy might be caused by cultural differences between Western countries and Oriental countries. Further research, however, is needed to account for the observed difference. Previous studies have suggested that the support of family members is also important (Hann et al, 1995), but we found that the physician plays the strongest role in patients' mental adjustment to cancer. Satisfaction with confidants as well as support from physicians are predictive of fighting spirit. It is noteworthy that not only the types of support but also patients' perception of support are related to their mental adjustment. These associations are consistent with those found in a previous study of cancer patients, which revealed that social support is a significant predictor of coping response (Bloom, 1982).

Age, education and household size were the sociodemographic variables predictive of helplessness/hopelessness as a mental adjustment. Older patients, less educated patients and patients living alone may respond worse to having cancer, and these patients may thus need additional intervention, such as psychotherapy, in order to cope better with cancer. Similar results were obtained in the analysis of medical characteristics. Interestingly, among medical characteristics only, performance status was significantly related to both helplessness/hopelessness and fighting spirit. Thus, cancer site per se may not affect patients' mental adjustment, but the physical impairment resulting from cancer does seem to affect their adjustment. Among the types of confidants, no variable was significantly associated with helplessness/hopelessness. In contrast, satisfaction with confidants was negatively correlated with helplessness/hopelessness, which is consistent with the positive correlation with fighting spirit.

In conclusion, the present study revealed that certain demographic, medical and social support variables are associated with certain styles of mental adjustment to cancer. In particular, performance status, the support of a physician and patients' satisfaction with confidants seem to affect whether the adjustment is adaptive or maladaptive. However, the results of this study may be questioned due to its lack of assessment of the content of the patients' confiding. The support of a physician, however, may help a cancer patient to adapt to having the disease, and a partnership attitude between the patient and physician seems to be crucial in several respects in improving the quality of life.

The limitations of this study include sampling bias (namely the study sample might not have been representative of Japanese cancer patients) and the cross-sectional design, both of which create many problems in attempts to assess causality. Further studies of the mental adjustment of cancer patients are needed to address remaining questions.

### ACKNOWLEDGEMENTS

The authors would like to acknowledge the collaborative support of the physicians and nursing staff at the National Cancer Center Hospital East and to thank Satoshi Sasaki MD, PhD, Epidemiology and Biostatistics Division, National Cancer Center Research Institute East, for his helpful statistical advice. This work was supported in part by a Grant-in-Aid for Cancer Research and Second-term Comprehensive 10-year Strategy for Cancer Control from the Japanese Ministry of Health and Welfare.

### REFERENCES

Akechi T, Kugaya A, Okamura H, Mikami I, Nishiwaki Y, Fukue M, Yamawaki S and Uchitomi Y (1997) Validity and reliability of the Japanese version of the Mental Adjustment to Cancer (MAC) scale (in Japanese). *Jpn J Psychiatr Treatment* **12**: 1065–1071

Bloom JR (1982) Social support, accommodation to stress and adjustment to breast cancer. *Soc Sci Med* **16**: 1329–1338

Bukberg J, Penman D and Holland JC (1984) Depression in hospitalized cancer patients. *Psychosom Med* **46**: 199–212

Cella DF, Orofiamma B, Holland JC, Silberfarb PM, Tross S, Feldstein M, Perry M, Maurer LH, Comis R and Orav EJ (1987) The relationship of psychological distress, extent of disease, and performance status in patients with lung cancer. *Cancer* **60**: 1661–1667

Cohen S (1988) Psychosocial models of the role of social support in the etiology of physical disease. *Health Psychol* **7**: 269–297

Dean C and Surtees PG (1989) Do psychological factors predict survival in breast cancer? *J Psychosom Res* **33**: 561–569

Di Clemente RJ and Temoshok L (1985) Psychological adjustment to having cutaneous malignant melanoma as a predictor of follow-up clinical status. *Psychosom Med* **47**: 81

Dunkel-Schetter C, Feinstein LG, Taylor SE and Falke RL (1992) Patterns of coping with cancer. *Health Psychol* **11**: 79–87

Evans DR, Thompson AB and Browne GB (1993) Factors associated with the psychological well-being of adults with acute leukemia in remission. *J Clin Psychol* **49**: 153–160

Ferrero J, Barreto MP and Toledo M (1994) Mental adjustment to cancer and quality of life in breast cancer patients: an exploratory study. *Psycho-oncology* **3**: 223–232

© Cancer Research Campaign 1998

Friedman LC, Nelson DV, Baer PE, Lane M, Smith FE and Dworkin RJ (1992) The relationship of dispositional optimism, daily life stress, and domestic environment to coping methods used by cancer patients. *J Behav Med* **15**: 127–141

Grassi L, Rosti G, Lasalvia A and Marangolo M (1993) Psychosocial variables associated with mental adjustment to cancer. *Psycho-oncology* **2**: 11–20

Greer S and Watson M (1987) Mental adjustment to cancer: its measurement and prognostic importance. *Cancer Surveys* **6**: 439–453

Greer S, Moorey S and Watson M (1989) Patients' adjustment to cancer: the mental adjustment to cancer (MAC) scale vs clinical ratings. *J Psychosom Res* **33**: 373–377

Greer S, Morris T, Pettingale KW and Haybittle JL (1990) Psychological response to breast cancer and 15-year outcome. *Lancet* **335**: 49–50

Hann D, Oxman T, Ahles TA, Furstenberg CT and Stuke TA (1995) Social support adequacy and depression in older patients with metastatic cancer. *Psycho-oncology* **4**: 213–221

House JS, Landis KR and Umberson D (1988) Social relationships and health. *Science* **241**: 540–545

Lampic C, Wennberg A, Schill JR, Glimelius B, Brodin O and Sjoden PS (1994) Coping, psychosocial well-being and anxiety in cancer patients at follow-up visits. *Acta Oncol* **33**: 887–894

Lansky SB, List MA, Herrmann CA, Ets-Hokin EG, DasGupta TK, Wilbanks GD and Hendrickson FR (1985) Absence of major depressive disorder in female cancer patients. *J Clin Oncol* **3**: 1553–1560

Maguire P, Booth K, Elliott C and Jones B (1996) Helping health professionals involved in cancer care acquire key interviewing skills – the impact of workshops. *Eur J Cancer* **32**A: 1486–1489

Maunsell E, Brisson J and Deschenes L (1995) Social support and survival among women with breast cancer. *Cancer* **76**: 631–637

Moorey S and Greer S (1989) *Psychological Therapy for Patients with Cancer: a New Approach.* Heinemann: London

Morris T, Pettingale K and Haybittle J (1992) Psychological response to cancer diagnosis and disease outcome in patients with breast cancer and lymphoma. *Psycho-oncology* **1**: 105–114

Neuling SJ and Winefield HR (1988) Social support and recovery after surgery for breast cancer: frequency and correlates of supportive behaviors by family, friends and surgeon. *Soc Sci Med* **27**: 385–392

Razavi D, Delvaux N and Hopwood P (1997) Improving communication with cancer patients. A challenge for physicians. *Annals NY Acad Sci* **809**: 350–360

Revenson TA, Wollman CA and Felton BJ (1983) Social supports as stress buffers for adult cancer patients. *Psychosom Med* **45**: 321–331

Roberts C, Cox CE, Shannon VJ and Wells NL (1994) A closer look at social support as a moderate of stress in breast cancer. *Health Soc Work* **19**: 157–164

Schnoll RA, Mackinnon JR, Stolbach L and Lorman C (1995) The relationship between emotional adjustment and two factor structure of the Mental Adjustment to Cancer (MAC) scale. *Psycho-oncology* **4**: 265–272

Schwartz CE, Daltroy LH, Brandt U, Friedman R and Stolbach L (1992) A psychometric analysis of the Mental Adjustment to Cancer Scale. *Psychol Med* **22**: 203–210

Slevin ML, Nichols SE, Downer SM, Wilson P, Lister TA, Arnott S, Maher J, Souhami RL, Tobias JS, Goldstone AH and Cody M (1996) Emotional support for cancer patients: what do patients really want? *Br J Cancer* **74**: 1275–1279

Stanton LA and Snider PR (1993) Coping with a breast cancer diagnosis: a prospective study. *Health Psychol* **12**: 16–23

Thomas SF and Marks DF (1995) The measurement of coping in breast cancer patients. *Psycho-oncology* **4**: 231–237

Wagner MK, Armstrong D and Laughlin JE (1995) Cognitive determinants of quality of life after onset of cancer. *Psychol Rep* **77**: 147–154

Watson M, Greer S, Blake S and Shrapnell K (1984) Reaction to diagnosis of breast cancer – relationship between denial, delay and rates of psychological morbidity. *Cancer* **53**: 2008–2012

Watson M, Greer S, Young J, Inayat Q, Burgess C and Robertson B (1988) Development of a questionnaire measure of adjustment to cancer: the MAC scale. *Psychol Med* **18**: 203–209

Watson M, Greer S and Bliss JM (1989) *Mental Adjustment to Cancer Scale User's Manual.* Cancer Research Campaign Medical Research Group, Royal Marsden Hospital: Sutton, Surrey

Watson M, Greer S, Rowden L, Gorman C, Robertson B, Bliss JM and Tunmore R (1991) Relationships between emotional control, adjustment to cancer and depression and anxiety in breast cancer patients. *Psychol Med* **21**: 51–57

Wortman CB (1984) Social support and the cancer patient – conceptual and methodological issues. *Cancer* **53**: 2339–2360

© Cancer Research Campaign 1998